# Functional Network Connectivity Patterns between Idiopathic Generalized Epilepsy with Myoclonic and Absence Seizures

**DOI:** 10.3389/fncom.2017.00038

**Published:** 2017-05-23

**Authors:** Qifu Li, Yongmin Chen, Yong Wei, Shengmei Chen, Lin Ma, Zhiyi He, Zhibin Chen

**Affiliations:** ^1^Department of Neurology, First Affiliated Hospital of Hainan Medical UniversityHaikou, China; ^2^Department of Neurology, First Hospital of China Medical UniversityShenyang, China; ^3^Department of Radiology, Maternal and Child Health Care Hospital of Hainan ProvinceHaikou, China

**Keywords:** absence epilepsy, functional network connectivity, fMRI, myoclonic epilepsy

## Abstract

The extensive cerebral cortex and subcortical structures are considered as the major regions related to the generalized epileptiform discharges in idiopathic generalized epilepsy. However, various clinical syndromes and electroencephalogram (EEG) signs exist across generalized seizures, such as the loss of consciousness during absence seizures (AS) and the jerk of limbs during myoclonic seizures (MS). It is presumed that various functional systems affected by discharges lead to the difference in syndromes of these seizures. Twenty epileptic patients with MS, 21 patients with AS, and 21 healthy controls were recruited in this study. The functional network connectivity was analyzed based on the resting-state functional magnetic resonance imaging scans. The statistical analysis was performed in three groups to assess the difference in the functional brain networks in two types of generalized seizures. Twelve resting-state networks were identified in three groups. Both patient groups showed common abnormalities, including decreased functional connectivity in salience network (SN), cerebellum network, and primary perceptional networks and decreased connection between SN and visual network, compared with healthy controls. Interestingly, the frontal part of high-level cognitive resting-state networks showed increased functional connectivity (FC) in patients with MS, but decreased FC in patients with AS. Moreover, patients with MS showed decreased negative connections between high-level cognitive networks and primary system. The common alteration in both patient groups, including SN, might reflect a similar mechanism associated with the loss of consciousness during generalized seizures. This study provided the evidence of brain network in generalized epilepsy to understand the difference between MS and AS.

## Introduction

The idiopathic generalized epilepsy (IGE) characterized by bilateral synchronous epileptic discharge (such as spike and wave discharges, SWD) in ictal or interictal electroencephalography (EEG) recording, which reflects abnormal oscillations in the corticothalamic network. It includes a group of epilepsy syndromes clinically characterized by generalized tonic–clonic seizures (GTCS), myoclonic seizures (MS), and absence seizures (AS) (Genton et al., 2013). As the most common childhood epilepsy syndrome (10–17%) of childhood-onset epilepsy (Crunelli and Leresche, 2002; Tenney and Glauser, 2013), typical AS present as brief episodes of staring and unresponsiveness, often accompanied by 2.5–4-Hz generalized SWDs in scalp EEG. The duration of seizures is usually < 10 s, and seizures can occur up to hundreds of times per day. Patients with MS are characterized by jerks, tonic–clonic seizures, and less frequently, AS (Janz, 1985). Standard interictal EEG features of patients with MS consist of 4–6-Hz generalized spike-wave or polyspike-wave discharges with a frontocentral predominance (Janz, 1985). In some studies based on neuroimaging, altered functional features in cortical and subcortical regions were observed in patients with AS or MS, suggesting the fundamental alteration in the brain to match the clinical syndromes. Despite remarkable distinction of clinical signs between two types of generalized seizures (AS and MS), the difference in their brains was not investigated clearly.

Recently, a rapidly increasing body of network analyses of structural and functional neuroimaging data has been used in the neuropsychiatric diseases such as schizophrenia (Chen et al., 2015, 2017), bipolar disorders (Dong et al., 2017), aging disease (Tan et al., 2015; Cao et al., 2016; He et al., 2017), and epilepsy (Liao et al., 2010; Luo et al., 2011b, 2014a; Ji et al., 2017). It provided the compelling evidence supporting that epilepsy would be described as a network disorder (Berg et al., 2010). It has been presumed that impairment of consciousness during generalized seizures is caused by a disrupted interaction among cortical and subcortical systems (Blumenfeld and Taylor, 2003; Blumenfeld, 2005, 2012; Cavanna and Monaco, 2009). Impaired cognition, memory function, and attentional deficits, as a long-term outcome of epilepsy, are also observed in these patients (Caplan et al., 2008). Moreover, interictal epileptic discharges also lead to the transient cognitive impairment (Hommet et al., 2006). Using functional magnetic resonance imaging (fMRI), many of the studies on focal epilepsy demonstrated the abnormality in subcortical network, default mode network, hippocampal network, and so on Liao et al. (2011) and Luo et al. (2015). Similarly, the altered functional networks covering cortical and subcortical structures were observed in patients with IGE (Luo et al., 2012a; Dong et al., 2016). In these networks, the corticothalamic network would be a key network to be studied extensively in IGE because it contributes to the generation and propagation of generalized SWD. Further, the abnormalities in default mode network (Luo et al., 2011a), attention network (Killory et al., 2011; Yang et al., 2013; Li et al., 2017), and salience network (SN) (Luo et al., 2014b) were also observed in patients with AS. Different from AS, the frontothalamic network and motor system demonstrated the abnormalities in patients with MS, suggesting the relationship with the frontocentral high-amplitude SWDs (Dong et al., 2016; Jiang et al., 2016). Interestingly, the hyperconnectivity in the motor system, including motor-related cortex and cerebellum, was observed in patients with MS. Thus, AS and MS would influence the different brain networks. The interaction among networks might be an appropriate choice to evaluate the difference between MS and AS. As an extension of functional connectivity (FC), functional network connectivity (FNC) was developed to characterize interactions distributed among different networks (Jafri et al., 2008). In previous studies, disturbed FNC has been observed in schizophrenia (Jafri et al., 2008) and epilepsy (Luo et al., 2012b). It was hypothesized that the FNC among brain networks, especially those related to brain baseline function and consciousness such as SN, would be disturbed in childhood absence epilepsy.

Twenty-one AS patients without MS and 20 patients with MS were recruited in the present study to evaluate the FNC based on resting-state fMRI. Moreover, the association between the altered FNC and clinical features was analyzed.

## Materials and methods

### Subjects

Forty-one patients were diagnosed with IGE based on the clinical and seizure semiology information consistent with the International League Against Epilepsy guidelines (Engel, 2001) by neurologists (QL and ZC). Twenty epileptic patients with MS and GTCS (13 females, age: 18.2 ± 6.3 years; disease duration: 11.1 ± 5.6 years) were categorized as MS group. Twenty-one patients (12 females, age: 12 ± 3.4 years) with AS were categorized as AS group, in which GTCS was observed in six patients with AS. No structural abnormality was observed in the routine brain neuroimaging including computed tomographic scanning and magnetic resonance imaging (MRI). Twenty-one healthy subjects were recruited as a sex-matched control group (HC) (12 females, age: 18.3 ± 5.6 years). All the controls were free of neurological or psychiatric disorders. This study was approved by the ethical committee of the First Affiliated Hospital of Hainan Medical University according to the standards of the Declaration of Helsinki. Written informed consent was obtained from each subject.

### Data acquisition

All patients and HC underwent MRI scanning in a 3T SIEMENS scanner with an eight-channel-phased array head coil (MAGNETOM Spectra, Siemens Healthcare, Erlangen, Germany) in the Maternal and Child Health Care Hospital of Hainan Province. The resting-state functional data were collected using an echo-planar imaging sequence with the following parameters: repetition time (TR), 2,000 ms; echo time (TE), 30 ms; flip angle (FA), 90°; field of view (FOV), 24 × 24 cm^2^; matrix, 64 × 64; and slice thickness, 4 mm with 0.4-mm gap, and 255 volumes in each run. Axial anatomical T1-weighted images were acquired using a three-dimensional fast spoiled gradient echo sequence [TR, 6.012 ms; TE, 1.968 ms; FA, 9°; matrix, 256 × 256; FOV, 25.6 × 25.6 cm^2^; slice thickness, 1 mm (no gap)] to generate 152 slices. All subjects were instructed to be “relaxed, eyes closed” and kept awake during the scanning.

### Data preprocessing

Preprocessing of fMRI dataset was conducted using the SPM8 software package (statistical parametric mapping available at: http://www.fil.ion.ucl.ac.uk/spm). The first five volumes of each run were discarded to ensure magnetic field stabilization. Any subject with head motion exceeding 2 mm and/or 2°C was excluded. The realigned images were spatially normalized to the Montreal Neurological Institute template using a 12-parameter affine transformation and resliced with a voxel size of 3 × 3 × 3 mm^3^. Then, spatial smoothing with a Gaussian kernel (8-mm full width at half maximum) was performed. Finally, the signals of white matter, cerebrospinal fluid, and linear drift were regressed from the smoothed data.

### Independent component analysis

Group spatial independent component analysis (ICA) was applied to the regressed fMRI data of all 61 participants using GIFT software (available at: http://icatb.sourceforge.net/, version 2.0a). Data were decomposed into some independent components (ICs). The number of components was estimated using the description length criterion. First, all datasets were concatenated temporally forming a 2D Space^*^Concatenated time data matrix. Then, the principal component analysis was used to reduce data dimension. After that, the Infomax ICA algorithm was used for IC estimation. For each individual dataset, a dual regression analysis was used to identify spatial maps and associated time courses corresponding to the aggregated components.

The resting-state networks (RSNs) were selected using anatomical information according to previous studies. For each selected RSN, the full factorial analysis was performed to detect the FC difference between groups using the SPM8 software package (statistical parametric mapping available at http://www.fil.ion.ucl.ac.uk/spm). The group comparisons were restricted to the voxels within the RSNs. For each RSN, the mask was created by combining the regions of the RSN in all participants using a one-sample *t*-test (*P* < 0.05; corrected using family wise error (FEW) criterion).

### Functional network connectivity

The time course of each selected RSN was extracted, which represented the average signal of all the voxels within a given network. FNC of two networks meant the correlation between the time series representing those networks. A Cross-correlation was computed between all selected RSNs using the maximal lagged-correlation approach. The lag between time courses ranged from −5 to +5 s. The absolute maximal lagged Pearson's correlation was calculated. The correlation coefficients were Fisher z-shifted. In this way, FNC maps were computed for the three groups. Using one-sample *t*-test, statistically significant [*P* < 0.05, false discovery rate (FDR) corrected] connections were extracted for patients and healthy controls separately. One-way analysis of variance (ANOVA) and *post hoc* (Tukey–Kramer) tests were used to identify the difference in FNC between three groups.

## Results

### Identification of RSNs

**Figure 1:**
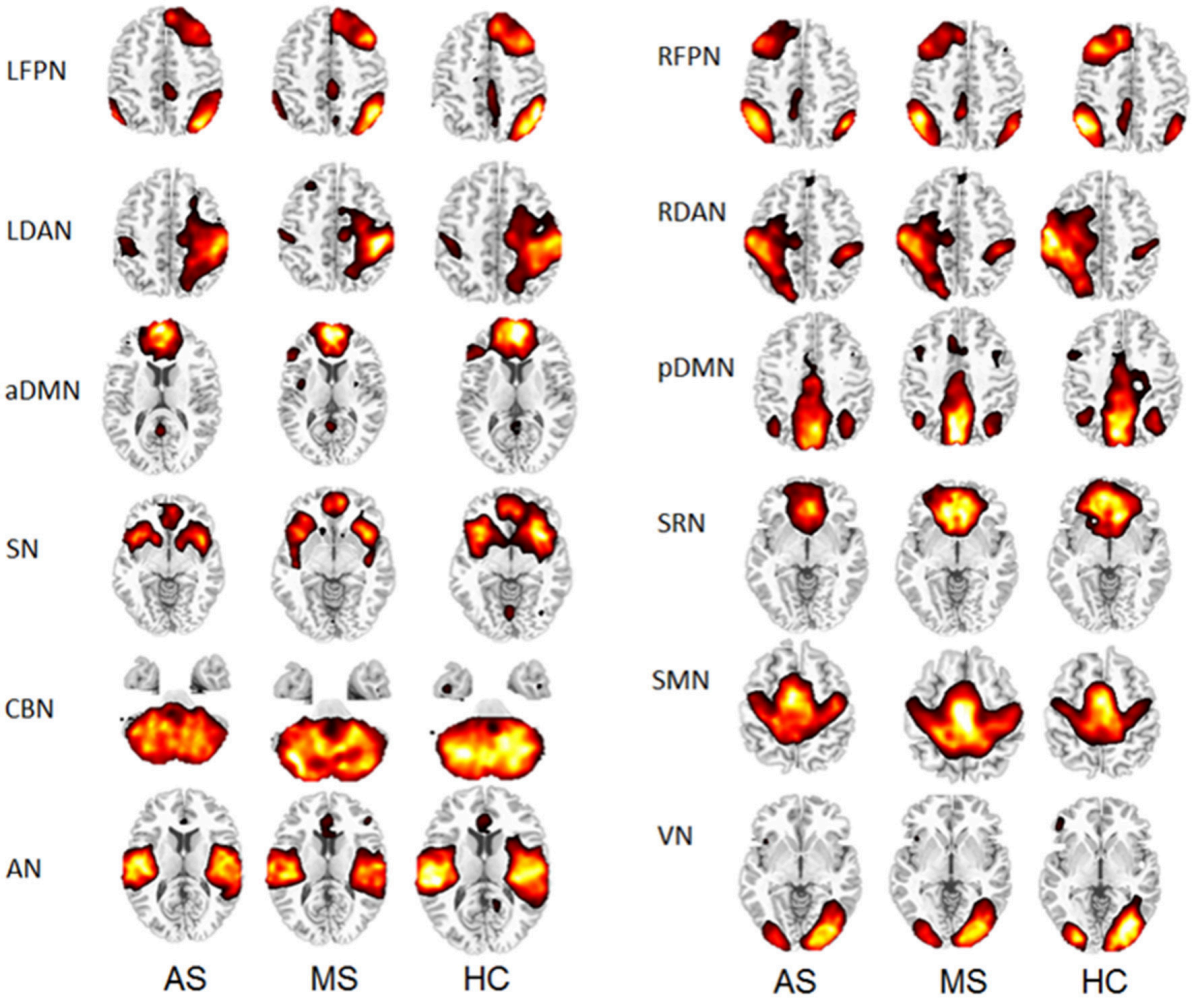
**Twelve RSNs in three groups**.

Thirty-four ICs were obtained from the group ICA. Twelve RSNs were selected for this study, including left frontoparietal network (LFPN), right frontoparietal network (RFPN), left dorsal attention network (LDAN), right dorsal attention network (RDAN), anterior part of the default mode network (aDMN), posterior part of the default mode network (pDMN), self-referential network (SRN), SN, cerebellar network (CBN), sensorimotor network (SMN), auditory network (AN), and visual network (VN). The spatial maps of the 12 RSNs are shown in Figure 1 for three groups (HC, MS, and AS).

### Group comparisons of the RSNs

**Figure 2:**
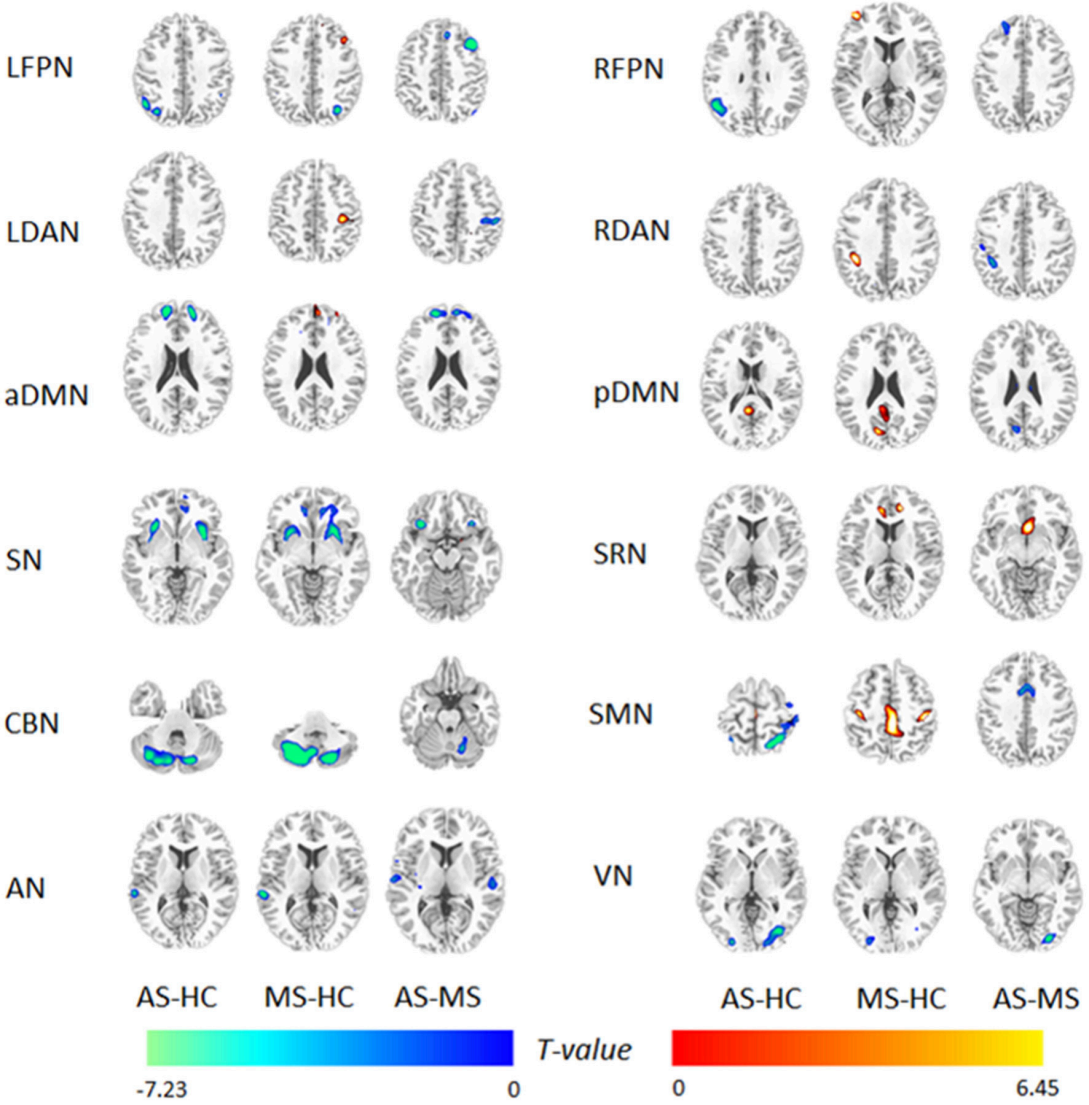
**Difference in functional integration in 12 RSNs between two groups**. AS–HC means that patients with AS had decreased FC compared with HC; MS–HC means that patients with MS had increased FC compared with HC; AS–MS means that patients with AS had decreased FC compared with patients with MS.

Using the full factorial analysis in SPM8, the difference in FC between groups was demonstrated in all 12 RSNs. Figure 2 illustrates the difference in FC between groups (*P* < 0.05, FDR corrected). In short, patients with MS showed an increased FC in the frontal region of high-level cognitive networks, including bilateral FPN, DAN, and aDMN, compared with HC. However, patients with AS demonstrated a decreased FC. In the SN and primary networks including CBN, VN, and AN, both patient groups showed decreased FC; the AS group showed more decreased FC. Besides, enhanced FC in SMN was observed in patients with MS rather than AS.

### FNC analysis within groups

**Figure 3:**
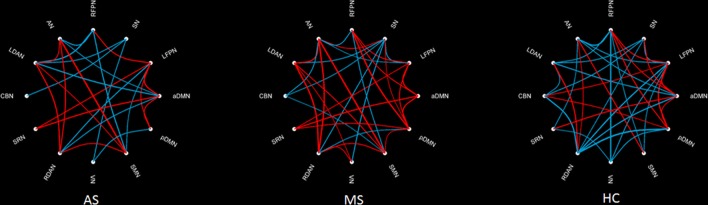
**Within-group FNC in three groups**. The positive correlation is represented by a red line, and the negative correlation by a blue line.

Figure 3 shows an FNC diagram for three groups (left, AS group; middle, MS group; and right, HC group). The positive correlation is represented in red, and the negative correlation in blue.

**Figure 4:**
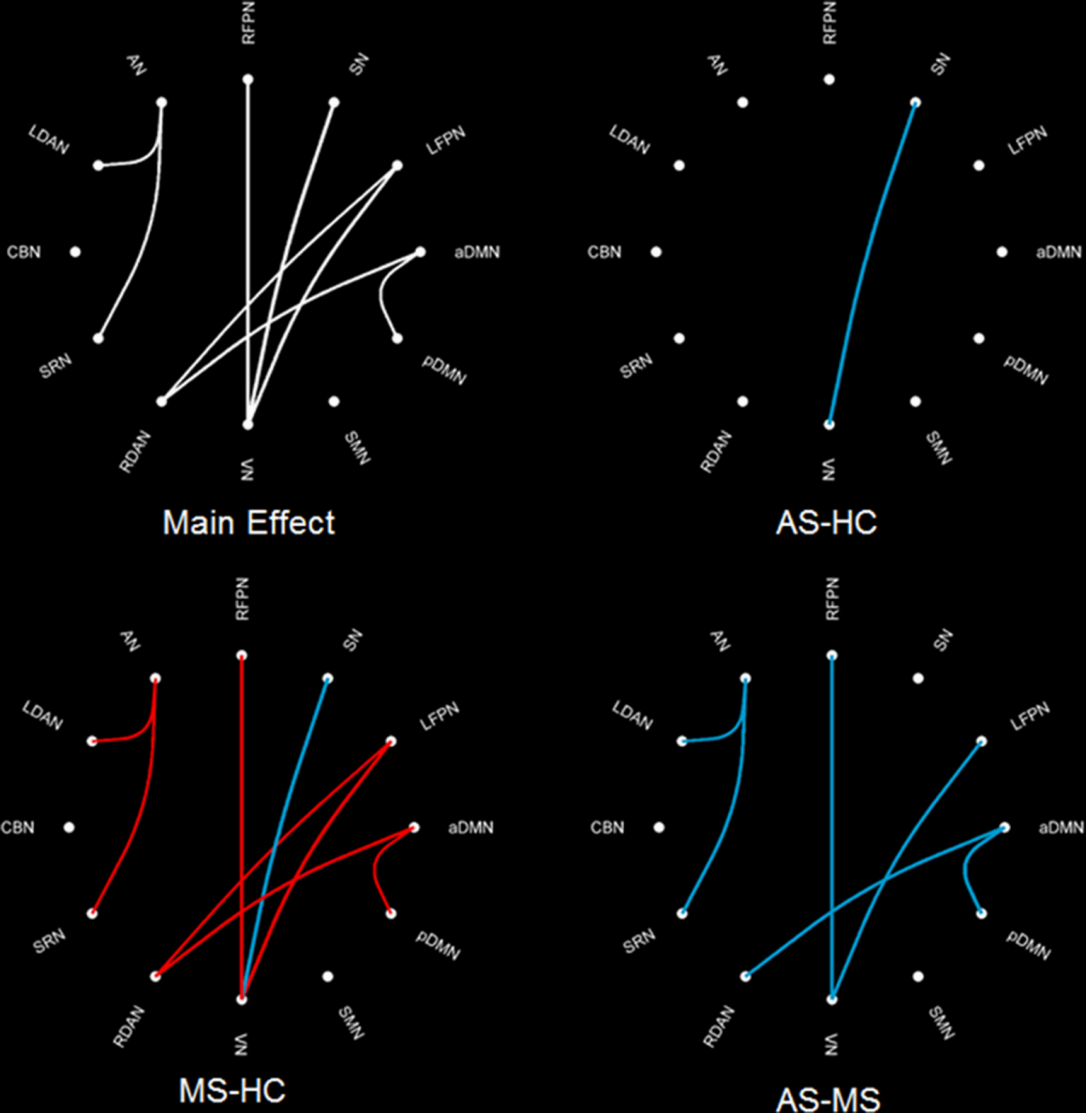
**Difference in FNC among three groups**. The main effect (left-up) means the result of ANOVA in three groups. MS–HC (left-bottom) means the *post hoc* difference between patients with MS and HC. Red line means that the FNC in MS is more than that in HC, and blue line means that the FNC in0020MS is less than that in HC. AS–HC (right-up) means the *post hoc* difference between patients with AS and HC (AS < HC). AS–MS (right-bottom) means the *post hoc* difference between patients with AS and MS (AS < MS).

The intergroup difference in FNC was investigated using one-way ANOVA and *post hoc* (Tukey–Kramer) tests. First, eight connections were found among RSNs with a significant group difference (*P* < 0.05, FDR corrected; Table 1), including five connections between high-level cognitive networks and visual (LFPN, RFPN, and SN) and auditory (RDAN and SRN) systems, two connections among high-level cognitive systems (LDAN link with aDMN and LFPN), and the connection between aDMN and pDMN. In a *post hoc* test, patients with MS showed increased interaction in all connections except for connections between VN and SN compared with HC. However, all patients with AS and MS showed decreased interaction between VN and SN compared with HC. Further, a decreased interaction between high-level cognitive networks and visual (LFPN and RFPN) and auditory (RDAN and SRN) systems, between aDMN and pDMN, and between aDMN and LDAN were observed in the AS group. The connection pattern is illustrated in Figure 4.

**Table 1:** **Significantly altered connections between functional networks in three groups**.

**RSN**	**RSN**	**ANOVA**	***Post hoc*** **(AS vs. HC)**		***Post hoc*** **(MS vs. HC)**		***Post hoc*** **(AS vs. MS)**	
		***P*-Value**	Mean diff.^*^	***P* Value**	Mean diff.^*^	***P* Value**	Mean diff.^*^	***P*-Value**
AN	RDAN	0.0006	0.0793	0.5161	0.2910	0.0005	−0.2117	0.0140
AN	SRN	0.0034	0.0446	0.8537	0.2787	0.0045	−0.2341	0.0196
VN	RFPN	0.0000	0.1270	0.1951	0.3673	0.0000	−0.2403	0.0050
VN	LFPN	0.0003	0.1456	0.2180	0.3766	0.0002	−0.2311	0.0276
VN	SN	0.0000	−0.3067	0.0003	−0.4330	0.0000	0.1263	0.2225
LDAN	LFPN	0.0031	0.0964	0.3887	0.2611	0.0023	−0.1647	0.0745
LDAN	aDMN	0.0021	0.0861	0.5713	0.3094	0.0019	−0.2233	0.0315
Pdmn	aDMN	0.0042	−0.0070	0.9962	0.2534	0.0116	−0.2604	0.0092

* *Difference between the two groups. For example, “0.0793” means the difference in averaged functional connectivity between AN and RDAN in the AS and HC groups (AS–HC)*.

## Discussion

Twelve RSNs in two epilepsy patient groups with AS and MS were identified in this study. Both patient groups showed common abnormalities, including decreased FC in SN, CBN, and primary perceptional networks and decreased connection between SN and VN, compared with HC. Interestingly, patients with MS showed more specific alteration than patients with AS. For example, the frontal part of high-level cognitive RSNs showed increased FC in patients with MS but decreased FC in patients with AS. Moreover, patients with MS showed decreased negative connections between high-level cognitive networks and primary system. The common alteration in both patient groups including SN might reflect a similar mechanism associated with the loss of consciousness during generalized seizures. The abnormality in the frontal lobe would be associated with the EEG sign: polyspikes and wave complexes with frontocentral predominance in patients with MS. Thus, this study provided the evidence of brain network in IGE to understand the difference between MS and AS.

In general, the SN, including the dorsal anterior cingulated cortex, bilateral anterior insula, and anterior temporoparietal junction (TPJ) (Seeley et al., 2007; Menon and Uddin, 2010; Kucyi et al., 2012), is involved in the detection of internal and external stimuli (Seeley et al., 2007; Menon and Uddin, 2010) and plays a fundamental role in awareness (Craig, 2009) and tonic alertness maintenance (Sadaghiani et al., 2010). Also, patients with AS showed deactivation related to epileptic discharges in extensive cortex including insula (Li et al., 2009). Previous studies demonstrated altered functional connection in SN in various consciousness states such as vegetative state and coma conditions (Vincent et al., 2007; Horovitz et al., 2008; Laureys and Schiff, 2012). Luo and colleagues showed reduced FC in SN in children with AS (Luo et al., 2014b). Although the simple MS dose not accompany with the loss of consciousness, all of the patients with MS in this study suffered from generalized tonic–clonic seizures apart from MS. Thus, the present finding, the significant alteration related to SN, would reflect interruption in the processing of external information and take part in the loss of consciousness during generalized seizures. Interestingly, the dysfunctional connection between VN and SN was also observed in the present study. The visual system is considered as a major input access of information in the central nervous system. The disconnection between VN and SN contributes to the unavailability to process external stimuli during seizures in IGE.

The decreased FC in the CBN was observed in both patient groups in this study. Recently, Kros and colleagues demonstrated that cerebellar nuclei served as an important modulator of the cerebral cortex during generalized seizures (Kros et al., 2015a). They also suggested the potential antiepileptic possibility of stimulation of cerebellar nuclei (Kros et al., 2015b). The decreased FC of CBN might imply the regulation of cerebral cortex by cerebellum in patients with AS or MS.

The most common EEG pattern in patients with MS includes the generalized irregular polyspike—wave complex with frontocentral accentuation. Previous studies showed abnormal frontal cortical gray matter volume and diffusion measures in patients with MS (O'muircheartaigh et al., 2011; Kim et al., 2012). Consistent with previous findings, the MS-specific alteration showed the increased FC in the frontal part of high-level cognitive RSNs and decreased FNC between frontal high-level cognitive networks and primary system in the present study. This finding suggested the abnormal function in the frontal lobe of patients with MS, which would be associated with the typical EEG sign: polyspike-wave complexes with frontocentral predominance. The high frequency of frontal executive dysfunction and abnormal verbal or visual memory was observed in patients with MS using neuropsychological testing (Pulsipher et al., 2009; Kim et al., 2012). Although the neuropsychological test was not performed in this study, it was presumed that the functional alteration in the frontal lobe might contribute to the cognitive disruption in patients with MS.

Patients with MS illustrated enhanced FC in SMN, while decreased FC in SMN was observed in the AS group. In patients with MS, the myoclonic jerks were presumed to have hyperexcitability in the motor-related regions in the frontal lobe. A previous study demonstrated structural and functional abnormalities in SMA and primary motor cortex in patients with MS (Vulliemoz et al., 2011). On the contrary, the predominant involvement of the somatosensory region in AS was reported in both animal (Polack et al., 2007; Polack and Charpier, 2009) and human studies (Blumenfeld, 2005; Yang et al., 2012; Xue et al., 2014). The various roles of SMN in MS and AS might contribute to the distinct alteration observed in the present study.

This study had several limitations. First, the age of onset of two types of seizures was different. The AS often occurred in patients during childhood, but MS occurred during adolescence. Thus, the mismatch in age between patient groups might have introduced the bias in the results. Second, the present study did not rule out the confounding effects of antiepileptic drug treatment. The contribution of drugs to FNC in the resting state still could not be identified. Third, generalized tonic–clonic seizures were observed in all patients with MS and six patients with AS; the same accompanied seizures would interrupt to identify the differences in both patient groups. Finally, the neuropsychological measurement was not performed in this study, leading to the inadequacy to interpret cognition-related dysfunction in FNC.

In conclusion, IGE patients with different seizures (AS and MS) have different EEG signs and clinical syndromes. The findings from the resting-state fMRI would provide some interesting evidence to assess the difference in extensive cerebral cortex and subcortical structures, even in the cerebellum. However, further investigations would be made in the future based on this preliminary study.

## Ethics statement

Ethical approval for this work was obtained from the Research Ethics Committee, the First Hospital of Hainan Medical University. All the subjects signed the informed consent.

## Author contributions

QL and YC collected the data and wrote the article; YW analyzed the data, SC critically revised the article; LM partially performed the experiments in Figures; ZC and ZH designed and guide the research.

### Conflict of interest statement

The authors declare that the research was conducted in the absence of any commercial or financial relationships that could be construed as a potential conflict of interest. The reviewer CL and handling Editor declared their shared affiliation, and the handling Editor states that the process nevertheless met the standards of a fair and objective review.
